# 

**Published:** 1832-06

**Authors:** 


### MONTHLY LIST OF MEDICAL BOOKS.

[*Medical Works cannot be entered on this List unless a copy be sent for the purpose, the titles of Books having frequently been sent to us as published which have not been published for weeks, or even months, after.*]

An Introduction to the Practice of Midwifery. By the late Thomas Denman, M.D. Licentiate in Midwifery of the College of Physicians, London; and Honorary Member of the Royal Medical Society at Edinburgh. Seventh Edition, with additional Modern Information on the various Subjects treated of in the Work. To which is added, a Dissertation on the Transfusion of Blood in the more dangerous Varieties of Uterine Hæmorrhage. By Charles Waller, M.D., Consulting Accoucheur to the London and Southwark Midwifery Institution, Lecturer on Midwifery, &c.—8vo. E. Cox, London.

A Clinical Report of the Royal Dispensary for Diseases of the Ear: with Remarks on the Objects and Utility of the Institution. By J. Harrison Curtis, Esq., Aurist in Ordinary to his Majesty, &c.—8vo. pp. 50. Longman, London.

Mr. Curtis's Dispensary deserves the support of the charitable and wealthy. Since it was instituted, upwards of ten thousand patients have been admitted, of whom nearly one half are reported as cured, and a large proportion have been greatly relieved.

A Practical Treatise on the Forms, Causes, Sanability, and Treatment of Pulmonary Consumption. By Edward Blackmore, M.D., Physician to the Plymouth Public Dispensary. —8vo. pp. 242. Longman, London.

Observations on Surgery and Pathology; illustrated by Cases, and by the Treatment of some of the most important Surgical Affections. By Wm. James Clement, Surgeon.— 8vo. pp. 230. Whittaker, and Highley, London.

The Anatomy of the Thymus Gland. By Sir Astley Cooper, Bart. F.R.S., Sergeant Surgeon to the King, Consulting Surgeon to Guy's Hospital, &c.—4to. pp. 47; five Plates. Longman, London.

The Cyclopædia of Practical Medicine. Edited by John Forbes, M.D., A. Tweedie, M.D., and John Conolly, M.D. Part V., for May.—Royal 8vo. Sherwood and Co. London.

Part VII. The Principles and Practice of Obstetric Medicine, in a Series of Systematic Dissertations on Midwifery, and on the Diseases of Women and Children. Illustrated by numerous Plates. By David D. Davis, M.D., M.R.S.L., Professor of Midwifery in the University of London, &c.—4to. John Taylor, London.

On the Structure of the Human Placenta, and its Connexion with the Uterus. By Robert Lee, M.D. F.R.S. &c., Physician to the British Lying-in Hospital. (From the Philosophical Transactions.)—Richard Taylor, London.

### APOTHECARIES' HALL.

Names of Gentlemen to whom the Court of Examiners have granted Certificates:

MAY 2.

Henry Peareth Brumell, Morpeth
Samuel Wright Fearn, Derby
Thomas Johnston, ditto
Richard Lanyan, Cambridge
Jonas Leake, Kilrush, Ireland
Samuel Mason, Olney
Richard Mallam, Oxford
Stephen Henry Pade, Devonport
Frank Dobson Potter, Ongar
Henry Glasford Potter, Newcastle on Tyne
Robert Rendell, Bradford, Somerset.

MAY 10.

John Chatto, no address
William Major, of Hungerford, Berks.
John Hutchinson Norris, Bury

John Stephens Phillott, Somersetshire
Henry Daniel Shea, Portsmouth
Ezekiel George Varenne, London
Frederick Worthington, Liverpool

MAY 17.

Walter R. H. Barker, no address
Henry Franklin Foley, of Durham
Edward Gee, Bristol
William Harding, Ludlow
Wm. Bakewell Matthews, Ashby de la Zouch
Wm. Henry Power, no address
George Miller Prichett, Yeovil
Edward Tippetts, Dartford

MAY 24.

Arthur Abraham Brett, of Wisbeach
Augustus Frederick Chalk, Dover
William Case, Portsmouth
Issaac Flower (of Knook, Wilts.), London
George Horton, Bromsgrove
Thomas Pedder Hutton, Beetham
Robert Hope Jones, Conway
William Jawell Roper, Norwich
Thomas Sadler, Cheltenham
Thomas Tweddle, Longtown
Charles Westron, Wellington
William Wheeler, Leominster
Joseph Walker, Belper.

### METEOROLOGICAL JOURNAL,

TO CORRESPONDENTS.

DR. RIDGWAY's *Paper will be inserted in the next Number.*

*The continuation of* DR. CLENDINNING's *Paper has been received.*

ERRATUM in the last Number of our Journal.—In the foot note, page 356, for Opium ℥i. read ʒi.

### INDEX

### TO THE TWELFTH VOLUME OF THE NEW SERIES

OF

### THE LONDON MEDICAL AND PHYSICAL JOURNAL.

PAGE

Abdominal abscess, case of 165, 166
Abortion, most frequent cause of, is in the product of the conception itself 231
Abscess, cases of abdominal . 165, 166
Abstinence, effects of . . 233
Africa, Medico-Historical Account of the Western Coast of . . 224
Air, similarity of atmospheric, in all situations . . . 316
Alum a cure for toothach . 427
Amenorrhœa, different causes of . 217
Anasarca . . . . . 238
Anatomical model, M. Auzoux's . 436
Aneurism, femoral; external iliac tied with success . . . 1
  inguinal; external iliac tied with success . . 76
  iliac, external iliac tied with success . . 32
  case of circumscribed brachial, cured . . 384
Angina pectoris, results in many cases relative to the existence of organic disease in general in . . 239
  cause of pain in . 240
Ani, division of sphincter . 429
Anæmia, description and cases of . 236
Apothecaries' Hall: names of gentlemen who have received certificates . 525
Arteries: experiments for the purpose of shewing that arteries may be obliterated without ligature, compression, or knife . . . 324
  on the ligature of . 214
Artery, external iliac, tied with success, 1, 32, 76
Asphyxia, phenomena of . . 402
  theory of . . 405
  cases of . . 332
Asthma no more than a symptom . 159
  convulsive, cured by carbonate of iron . . 416
  pathology of . . 409
  diagnosis of . . 412
  treatment in the paroxysm 413
Axilla, free opening necessary in abscess of, 428

Belladonna, poisoning by . . 514
Beulah Saline Spa at Norwood . 244

PAGE

Bile ducts, great dilatation of . 169
Bishop, the murderer: appearances on examining his body . . 83
Blood, critical and experimental essay on the circulation of . . 129
  standard analysis of healthy 392
  microscopical researches on, in disease . . . 424
  microscopical observations upon, in its pathological state 168
Bone digester, Papin's improved . 432
Botany, Professor Burnett's Lecture on 364
Brain, disease of, with paralysis . 480
Burking system, on the . . 81

Calculus, a sacculated, left with safety in the bladder . . . 127
  in the bladder, formed by a needle which had been swallowed, and remained in the bladder for a year 245
Calomel combined with nitre produces no salivation . . . 169
Cancer cured by excision and diet of Indian corn . . . 275
Capillaries, irritability of, doubted 135
Chlorate of potash recommended in cases of prurigo pudendi . . 427
Chlorosis, electricity recommended in, by Dr. Gooch . . . 216
Cholera in London: cases, &c. 258, 345
  order from the Privy Council relating to the . . 349
  Asphyxia . . 414
  description of . . 43
  distinction between mild and severe . . . 46
  proximate cause of . 47
  utility of opium in . 48
  on bleeding in . . 49
  influence of localities on the prevalence of . 51
  on predisposition to . 52
  treatment of . 67, 68
  notice of various works on 321
  on the chemical pathology of malignant . 389
  progress of the malignant, in England and Scotland . 255
  on the external use of opium in 355

Cæsarean operation successfully performed 170
Colchicum, poisoning by . . 515
Cold as a cause of disease, remarks on 439
morbific properties of . 443
Contagion from opening a grave . 423
on the evidence of . 497
Cornea, on interlaminary opacities of the 358
Cubebs, cases of eruptive disease from the use of . . . 195
Cuvier, death of . . . 524
Cyclopædia of Practical Medicine: analysis of Part I. . . . 230

Delirium Tremens, on the history and treatment of . . 98, 197, 280
from opium . 306
Dew, theory of . . . 314
Dispensing medicine, death from mistake in; verdict of manslaughter returned . . . . 524
Dropsy treated with elaterium . 381
Drunkenness, disease resembling . 468
Dysmenorrhœa, camphor recommended in, 218
Dyspnœa from external violence . 66

Elaterium, cases of dropsy treated with 381
Elbow-joint, excision of . . 512
Electro-magnetism . 432, 519
Empyema treated successfully by paracentesis . . . 73
Epidemical diseases, on the causes of, 182, 264
Epidemics attributed generally to the quality of the atmosphere . 489
Epidemic disease of stomach and bowels at Douglas . . 14

Fæces, vomiting of, from strictures of the ilium . . . 351
Fever, cases of, with eruption resembling measles . . . 292
Fistula in ano cured in a consumptive patient . . . 512
Frænum linguæ, occasional hazard in division of . . . 64
Funis, management of presentation of 223

Galvanic action, to protect copper sheathing of vessels against corrosive action of sea-water . . 314
battery, description of largest 80
Gangrene, hospital, nature of . 428
Germs, incubation of morbific . 337
Gout, &c., tobacco employed as a local application in . . . 427

Head, cases of injuries of . . 59
Heart, on polypi of the, as an idiopathic affection . . . 53
disease of the, simulated by derangement of the liver . . 71
polypi of the, case of 147, 150
Hemorrhage, on pulmonary, by Dr. Watson . . . 249
Hemorrhoids, on tying . . 427
Hernia, case of scrotal . . 38
Hydatids of uterus simulating pregnancy 329

Hydrophobia, occurring three months after the bite of a cat . . 328
Hydrocele, injection proper in cases of, even if the testes are enlarged . 37
new mode of detecting 170
Hypertrophy of the heart, classification of different kinds of . . 154
treatment 157
Hysteric (?) affection of the knee . 428

Ignatia amara, its medicinal uses . 113
Ileum, strictures of, with fæcal vomiting 351
Incubation of morbific germs . 337
Insanity, on the medical treatment of 300
bloodletting to be cautiously used in . . . 307
benefit of ice applied to the head 308
pustules produced on the scalp . 309
on the use of opium in . 310
on the use of arsenic in . 311
on the use of camphor in 312
severe cases of, frequently recover, 300
alterations of structure in 302
affections of abdominal viscera causing . . 305
displacement of the colon common in . . ib.
alleviated by acute diseases 333
Insects, curious case of pestiferous 163
Intermittent fever at the island of Erromanga, . . . 175
Iodine, on the use of, in scrofula, &c. 141
utility of, in syphilitic affections of the skin . . 472
various preparations of, for external and internal use . . 145
Iritis, on turpentine in . . 22
Italian boy, letter respecting the murder of . . . 81

Kava or Ava shrub, properties of . 110
Kidney, rupture of the . . 91
King's College, annual report, &c. 432
names of prize men at 521
examination at . ib.
Knee-joint, wound penetrating the 87
case of diseased . . 211

Labia pudendi, œdema of, in pregnancy 222
Labour, curious case of . . 79
Lactation, mischief of protracted 70
Laryngotomy and tracheotomy performed upon a patient attacked with œdematous angina, after the use of fumigations of the deuto-chlorate of mercury . . . 119
Larynx, description of the interior of the, as seen after attempted suicide, by Herbert Mayo, F.R.S. . 465
Leeches, mortality of, during a storm 80
Leucorrhœa, warm applications sometimes useful in . . 220
Lithotrity and lithotomy compared 502
Liver, derangement of the, simulating disease of the heart . . 71
Lungs, nervous debility of . 417

Malaria, Dissertations on, &c. . 487
Medico-Botanical Society, prize questions 435
Menorrhagia, three kinds of, described by Dr. Gooch . . . 219
Midwifery, Dr. Gooch's Lectures on 216
Morphia, endermic application of 169
Muscular tissue, calcareous deposit in 210
Musk, influence of, on vegetation 519

Nauclea Gambir, medical properties of 111
Nerves, anastomosis between 4th and 5th 323
Nitre and calomel combined produce no salivation . . . 169

Œdema of the labia pudendi, mistaken for the head of the child . 222
Opium externally used in cholera . 355
how to correct the unpleasant effects of . . 169
free exhibition of, in peritonitis 474
Optics . . . . . 432
Orbit, fatal case of wound of the . 298

Palmar arch, hemorrhage from wounding 263
Pancreas, case of diseased . 246
Paralysis, with disease of the brain 480
Pericarditis, anatomical characters of 153
Peritoneum, wounds of, not so dangerous as generally believed . 167
Peritonitis produced by sounding to discover stone in the bladder . 126
large doses of opium given in 474
Pharynx, tumor of the, in an infant, removed by ligature . . 431
Phlebitis, case of . . . 295
Planeriæ, habits and propagation of 318
Poisoning by belladonna . . 514
colchicum . . 515
Polypi of the heart sometimes an idiopathic disease . . . 53
case of 147, 150
Pneumogastric nerves, anomaly in 245
Polypus of the uterus, ligature not to be tightened when pain is produced 221
Pregnancy simulated by hydatids of the uterus . . . . . 329
Prurigo pudendi cured by a lotion of chlorate of potash . . 427
Puerperal mania, cases of . . 120
general conclusions drawn from twenty-five cases 125
monomania, case of 121
dementia, case of . 122
convulsions, Dr. Gooch's practice in . . 223
Pulmonary hemorrhage, no lesion detectible by dissection in the majority of cases of . . . 251

Rheumatism treated by the external use of morphia . . . 169
Rupture of the recto-abdominal fascia, exposing the abdominal cavity, yet admitting of delivery . . 430

Scrofula, &c. use of iodine in . 141
Shoulder, dislocation of, easily reduced 429
Skin, on absorption by the . 420
Spinal chord, on the anatomy of, by Herbert Mayo, F.R.S. . . 466
Spleen, notice of a work on the, by Mr. Mac Nolty . . . 242
on softening of the . 422
on the functions of the . 451
Stricture of the great intestine . 335
Stomach, epidemic disease of, at Douglas 14
Styptic, experiments with a new . 83
powder, a new . . 172
Styptics, Mr. C. Hawkins' experiments on the use of . . . 171
Surgical Apparatus, Atlas and Description of . . . 243

Tail of the eel, curious structure discovered in the, by Dr. Marshall Hall 139
Testicle, disease of the, mistaken for carcinoma . . . 208
Testis, Mr. Guthrie's Lecture on disease of 36
Tetanus from a puncture of the foot 297
Throat, wound of the . . 93
Thrombus, fatal case of puncturing a 295
Thymus gland, on the functions of 461
Sir Astley Cooper's description of . 482
Thyroid gland, on the functions of 457
Tic douloureux cured by the cyanuret of potassium . . . 75
Tobacco as a local application in gout, &c. 427
enema, poisoning with . 170
Toothach, nitric acid recommended for 78
cured by alum . . 427
Trephining sometimes necessary when no symptoms exist of compression of the brain . . . 61
Tumor expanding the spinal cord and nerves, without impairing sensation or motion . . . 429
Turpentine in cases of iritis . 22

Umbilical cord, ossification of . 430
University of London, names of the prize men at . . . 520
Urethra, stricture of . . 42
Urine, retention of, cases of, occurring simultaneously . . . 5
propriety of instantly introducing the catheter urged . . 9
with ulceration of the urethra and bladder, and abscess of the prostate 10
from inflammation and swelling of the prostate 13
Uterus, hydatids of, simulating pregnancy . . . . 329

Vagina, case of imperforate . 425
on certain discharges from, resembling gonorrhœa . 326

COLLECTANEA . . 71, 165, 245, 328, 420, 512
INTELLIGENCE . . 81, 171, 255, 345, 432, 520
METEOROLOGICAL REGISTER 86, 174, 262, 350, 438, 526

### CRITICAL ANALYSES.

An Essay on the Epidemic Cholera of India. By R. Orton, Esq. . 42
On Polypi of the Heart as an Idiopathic Affection. By W. Harty, M.D. 53, 147
Elements of Surgery. By Robert Liston, F.R.C.S. L. & E. . . . 58
A Critical and Experimental Essay on the Circulation of the Blood, &c. By Marshall Hall, M.D., F.R.S. E., &c. 129
Essays on the Effects of Iodine in Scrofulous Diseases. From the French of M. Lugol, by Dr. W. B. O'Shaughnessy 140
A Treatise on the Diseases of the Heart and Great Vessels, &c. By J. Hope, M.D. (concluded) . . 153
A Practical Compendium of Midwifery; being the Course of Lectures on Midwifery, and on the Diseases of Women and Infants, delivered at St. Bartholomew's Hospital, by the late Robert Gooch, M.D. Prepared for publication by George Skinner, M.R.C.S. . 221
A Practical Medico-Historical Account of the Western Coast of Africa. By James Boyle, M.C.S.L. . . 224
The Cyclopædia of Practical Medicine, edited by John Forbes, M.D. &c., Alexander Tweedie, M.D., and John Conolly, M.D. &c. Published in Monthly Parts . . 230, 402
Observations on the Medical Treatment of Insanity. By E. J. Seymour, M.D., Physician to St. George's Hospital, &c. 300
Elements of Chemistry, including the recent Discoveries and Doctrines of the Science. By Edward Turner, M.D. F.R.S. L. & E. &c. . . 313
A History of British Animals. By John Fleming, D.D. &c. . . 318
The Universal System. By George Field 319
Horæ Entomologicæ. By W. Sharp Macleay . . . ib.
The Philosophy of Zoology. By John Fleming, D.D. . . . ib.
Illustrations of Nature, by G. T. Burnett ib.
Report on the Chemical Pathology of the Malignant Cholera; containing Analyses of the Blood, Dejections, &c. of Patients labouring under that Disease in Newcastle and London, &c. By W. B. O'Shaughnessy, M.D. . 389
Thoughts on Cholera Asphyxia, by R. Bree, M.D. F.R.S. . . 402
The Anatomy of the Thymus Gland. By Sir Astley Cooper, Bart. . 482
Dissertations on Malaria, Contagion, and Cholera; explaining the Principles which regulate Endemic, Epidemic, and Contagious Diseases, with a View to their Prevention. By W. Aiton, M.D. 487
Lithotrity and Lithotomy compared; being an Analytical Examination of the present Methods of Treating Stone in the Bladder. By Thomas King, M.D. 502

### BIBLIOGRAPHICAL NOTICES.

Cholera; its Nature, Cause, and Treatment, and Prevention, clearly and concisely explained. By C. Searle, Esq. 66
Cholera; its Non-contagious Nature, and the best Means of arresting its Progress, &c. By T. M. Greenhow, Esq. ib.
How is the Cholera propagated? The Question considered, and some Facts stated. By an American Physician 67
An Essay on the Nature and Treatment of the Indian Pestilence, commonly called Cholera. By H. Penneck, M.D. ib.
Of Pestilential Cholera; its Nature, Prevention, &c. By J. Copland, M.D. ib.
Thoughts on the best Means of lessening the Destructive Progress of Cholera, &c. By Joshua Brookes, F.R.S. &c. ib.
Pathological Anatomy of the Brain, Spinal Chord, &c. By W. P. Cocks, Esq. 69
Remarks on the Subject of Lactation. By Edward Morton, M.D. . 70
Researches to establish the Truth of the Linnæan Doctrine of Animate Contagions. By Adam Neale, M.D. . 162
Parts I. II. III. IV. and V. The Principles and Practice of Obstetric Medicine. By David D. Davis, M.D. &c. 164, 326
A Synopsis of the Bones, Ligaments, Muscles, Blood-vessels, and Nerves of the Human Body. By W. S. Cox, Surgeon . . . 164
A Modern Anatomy of the Human Spleen. By P. Mac Nolty, Surgeon, &c. 240
An Atlas of Surgical Apparatus; being a Series of Delineations of the most important Mechanical Auxiliaries of Surgery. By H. T. Chapman, M.R.C.S. 241
A Brief Description of Surgical Apparatus; intended to accompany the above. By H. T. Chapman, M.R.C.S. . ib.
An Account of the Beulah Saline Spa at Norwood; containing a Description of its Medicinal Properties and Effects. By G. H. Weatherhead, M.D. . 242
Letters on the Cholera, by W. Ainslie, M.D. 321
Letter to the President of the Westmin. Med. Soc. on Cholera. By Dr. Webster ib.
The Laws and Progress of the Epidemic Cholera. By Thomas Hancock, M.D. ib.
Substance of a Lecture on Cholera. By D. Baird, M.D. . . ib.
Cholera; its Character and Treatment. By C. T. Thackrah . . ib.
A Series of Experiments performed for the purpose of shewing that Arteries may be obliterated without Ligature, Compression, or the Knife. By Benj. Phillips . . . . . 324
Cases of Insanity; with Medical, Moral, and Philosophical Observations and Essays upon them. By M. Allen, M.D &c. Part I. Vol. I. . 19

### Names *of* Authors *of whose Works, Observations, &c. a more or less detailed Account is given in this Volume.*

Adams, Francis, Esq. on the Causes of Epidemical Diseases . 182, 264
Ainslie, Whitelaw, M.D. &c., notice of his Letters on Cholera . . 321
Aiton, W. M.D., Dissertations on Malaria, &c. (analysis) . . 486
Allen, M. M.D., cases of Insanity, &c. 419
Andrew, J. M.D., cases of Uterine Hydatids, simulating Pregnancy . 329
Auzoux, M., description of his Anatomical Model . . . 436
Baird, Dr. notice of his Lecture on Cholera, 321
Barnett, Mr. A., cases of Cholera . 346
Bennett, G. Esq. F.L.S. &c., on the Medicinal Properties, &c. of the Kava Shrub, &c. . . . 110
account of the Intermittent Fever at the Island of Erromanga 175
Berard, M., case of great Dilatation of the Bile Ducts . . . 169
Bignardi, M., case of Anomaly in the Pneumogastric Nerves . . 245
Bonafous, M., Composition of his Styptic 172
Bonnet, M., on the Endermic Application of Morphia in Rheumatism . 169
Bow, Dr., on the External Use of Opium in Cholera . . . 355
Boyle, James, Esq., Medico-Historical Account of the Western Coast of Africa, &c. (analysis) . . 224
Bree, R. M.D. F.R.S., on Cholera Asphyxia 414
Brodie, B. Esq., Clinical Lecture on a Case of Diseased Knee, &c.; with Remarks on the Ligature of Arteries, and Secondary Hemorrhage . 211
Clinical Remarks . 428
Brookes, Joshua, F.R.S., notice of his Letter on Cholera . . 69
Burdach, M., on Combination of Nitre and Calomel . . . 169
Burnett, G. T., F.L.S. &c., Lecture on Botany, delivered at King's College 364
notice of his "Illustrations of Nature" . . . 319
Chapman, H. T. Esq., Atlas of Surgical Apparatus, &c.; with a separate Description, &c. . . . 243
Clendinning, Dr. John, on Cold as a Cause of Disease, &c. . . 439
Cocks, W. P., Surgeon, notice of his Pathological Anatomy of the Brain, &c. 69
Cooper, Sir Astley, Bart., on the Anatomy of the Thymus Gland (analysis) 482
Cooper, Bransby, Esq. F.R.S., case of Scrotal Hernia . . . 33
successful Case of Femoral Aneurism . . . 1
Copland, James, M.D., notice of his work on Cholera . . 68
Cox, W. S., Surgeon, notice of his Synopsis of the Bones, Ligaments, &c. 164
Cusack, Mr. on a case of Circumscribed Brachial Aneurism, cured by Compression and Digitalis 384
D'Arcet, M., his improvement of Papin's Bone Digester . . . 432
Davis, Dr. D., notice of his Principles of Obstetric Medicine . 164, 326
Donné, M, Microscopical Observations on the Blood in its pathological State 168
Microscopical Researches on the Blood . . . 424
Drais, Dr., description of his ingenious Optical Instrument . . 432
Elliotson, Dr. on Absorption by the Skin 420
Ewen, H. Esq. cases of Asphyxia . 332
Ewing, Dr. Alexander, case of Inguinal Aneurism . . . 76
Faraday, M., F.R.S., on Electro-magnetism . . . 432, 519
Fearn, Mr., on an Anastomosis between the Fourth and Fifth Nerves . 328
Fereday, Thomas, Esq., on Poisoning with Colchicum . . . 515
Ferguson, Dr. Thomas, case of Anomalous Labour . . . 78
Field, George, Esq., notice of his Universal System . . . 319
Fleming, John, D.D. &c., notice of his Philosophy of Zoology . ib.
History of British Animals (notice of) . . . 31
Fletcher, R. Esq., case of Peritonitis from long-continued Efforts to find the Stone before Operation . 126
Foote, John, Esq. jun., on the use of Turpentine in Iritis . . 22
Forbes, Dr. on Asthma . . 408
Fouquier, Prof. case of Laryngotomy, &c. 119
Frank, M., case of Fistula cured in a Consumptive Patient . . 512
Gooch, Dr. Robert, Lectures on Midwifery, edited by G. Skinner, Esq. 217
Goppert, Dr., on the Influence of Musk on Vegetation . . . 519
Grahl, Dr., case of Poisoning with a Tobacco Clyster . . 170
Greenhow, T. M., Esq., notice of his work on Cholera . . 67
Gregory, Dr. George, on the Incubation of Morbific Germs . . 337
Guéneau de Mussy, M., case of Diseased Brain, with Paralysis, &c. . 480
Guthrie, G. J., F.R.S., successful case of Iliac Aneurism . . 32
Clinical Lecture on Disease of Testis . . . 36
fatal case of Wound of the Orbit by a Tobacco-pipe . 298
Hall, Dr. Marshall, analysis of his Essay on the Circulation of the Blood, &c. 129
Halma-Grand, M. Experiments with his new Styptic . . . 83
Hancock, Dr. Thomas, notice of his Laws and Progress of Cholera . 321
Harty, Dr. Wm., analysis of his paper on Polypi of the Heart . 53, 147
Hackman, Dr., on Softening of the Spleen, 422

Hawkins, Cæsar, Esq., his Experiments on the use of Styptics in Hemorrhage 171
Hope, Dr. J., analysis of his Treatise on Diseases of the Heart . . 153
Howship, J. Esq., on Spasmodic Stricture of the Colon . . 335
Jolly, M., Cæsarean Operation successful 170
Kerr, David, Esq., case of Excision of the Elbow-joint . . 512
Key, Mr., fatal case of Puncturing a large Thrombus connected with the Vena Saphæna . . . 295
King, Dr. Thomas, Lithotrity and Lithotomy compared (analysis) . 502
Koestler, M. A. L., cases of Poisoning by Belladonna . . . 514
Kuhn, Dr., on Alum as a cure for Toothach, 426
Laidlaw, James, Esq., Surgical and Pathological Cases . . 5, 87
Lawrence, W. F.R.S., Diseased Pancreas 246
Lecanu, M., his analysis of healthy Blood 392
Liston, Robert, Esq., Review of his Elements of Surgery . . 58
Logan, R. Esq., cases in Surgery . 429
Logan, —, Esq., case in which a Needle was swallowed, and a year afterwards extracted from the Bladder . 245
Lombard, Dr., cases of Tic Douloureux treated successfully by Cyanuret of Potassium . . . 75
Lugol, M., his Essay on Iodine (analysis) 140
Lynn, Mr. Tetanus from Puncture of Foot, 297
Macdonald, Dr., cases of Puerperal Mania, 120
Macleay, Mr. W. S., notice of his Horæ Entomologicæ . . . 319
Mac Nolty, Mr., on the Human Spleen 242
Mayo, Herbert, F.R.S., on the Distribution of the Grey Matter in the Spinal Chord, &c. . . . 466
Description of the Interior of the Larynx, seen after attempted Suicide 463
on the Efficacy of Iodine in Syphilitic Affections of the Skin 472
his Letter respecting the Murder of the supposed Italian Boy 81
Morton, Dr. Edward, notice of his work on Lactation . . . 70
Neale, Dr. A., notice of his Researches to establish the Truth of the Linnæan Doctrine of Animate Contagion, &c. 162
North, John, F.L.S., cases of an Eruptive Disease arising from Cubebs 195
Orton, Mr., review of his work on Cholera, 42
O'Shaughnessy, Dr., analysis of his Translation of Lugol's Essay on Iodine, 140
analysis of his Report on the Chemical Pathology of the Malignant Cholera . 389
cases of Cholera . . 348
Oswald, H. R., Esq., Letter to Sir H. Halford, on an Epidemic Disease of the Stomach and Bowels at Douglas 14
Peirce, Dr. Leonard, case of Cancer cured by Excisions and Diet of Indian Corn 275
Penneck, Dr., notice of his work on Cholera, 68
Phillips, Mr. Benj., notice of his Experiments on Arteries, to shew that they may be obliterated without Ligature, Compression, or the Knife . 324
Puchett, M., on a Corrector of Opium 169
Rennes, Dr., on a Disease resembling Drunkenness . . . 468
Roberts, Dr., case of Strictures of the Ileum, &c. . . . 349
Roget, Dr., on Asphyxia . . 402
Rogers, Dr. D. L., cases in Surgery 165
Ryan, Dr. M., Nitric Acid recommended by him for Toothach . . 78
Searle, C., Esq., notice of the second Edition of his Work on Cholera . 66
Segalas, M., on a Diagnosis of Hydrocele, 170
Seymour, Dr., on the Medical Treatment of Insanity (analysis) . . 300
Insanity alleviated by acute Disease . . 333
Sharp, W., Esq., cases of Hemorrhage from Wounding the Palmar Arch 263
Skinner, G., Esq., edition of Dr. Gooch's Lectures on Midwifery . . 217
Stedman, Dr., on Imperforate Vagina, &c. 425
Stokes, Dr., on the Exhibition of Opium in large Doses in Peritonitis, &c. 474
Talrich, M., Experiments with his new Styptic . . . 83
Thackrah, Mr. C. T., notice of his work on Cholera . . . . 321
Thomas, Mr. W. J., on the Treatment of Interlaminary Opacities of the Cornea 358
Trousseau, M., on the Endermic Application of Morphia in Rheumatism 169
Turner, Dr. Edward, F.R.S., Elements of Chemistry (review) . . 313
Tuson, E. W., F.L.S., notice of his Dissector's Guide . . 437
Vaughan, Dr. J. F., case of Hepatic Derangement, simulating Organic Disease of the Heart, &c. . 71
Vetch, Dr., on use of Tobacco in Gout 427
Waller, Dr., half-yearly Midwifery Report, 96
Ware, Dr., on Delirium Tremens, 98, 197, 280
Watson, Dr., on Pulmonary Hemorrhage, 249
Weatherhead, Dr. G. H., account of the Beulah Spa at Norwood, Surrey 244
Weber's Anatomical Atlas, noticed 173
Webster, Dr. J., notice of his Letter on Cholera . . . . 321
Wendelstadt, Dr., account of his own Case of Empyema, successfully treated by Paracentesis . . . 73
Wilson, Dr., cases of Dropsy treated with Elaterium, &c. . . 381
cases of Fever with Eruption resembling Measles and early Debility . 292
case of Phlebitis . . 295
Wright, Dr., Disease of Testicle mistaken for Cancer . . 208
Calcareous Deposit in the Muscles, 210

